# Health and capabilities: a conceptual clarification

**DOI:** 10.1007/s11019-019-09902-w

**Published:** 2019-05-06

**Authors:** Per-Anders Tengland

**Affiliations:** Health and Society, Malmö University, Jan Waldenströms gata 25, 205 06 Malmö, Sweden

**Keywords:** Ability, Amartya Sen, Capabilities, Functionings, Health, Martha Nussbaum, Opportunity, Real possibility

## Abstract

There are great health disparities in the world today, both between countries and within them. This problem might be seen as related to the access to various kinds of capabilities. It is not fully clear, however, what the exact relation is between health and capabilities. Neither Amartya Sen nor Martha Nussbaum has explicitly formulated a theory of health to go with their theories of capabilities. This paper attempts to present a clarification of the conceptual relation between health and capabilities. Health, it is argued, should be seen as a holistic multi-dimensional phenomenon, made up of basic abilities and subjective well-being, and of fundamental states and processes. Using this theory, the paper shows how health is related to Nussbaum’s ten capabilities. It is argued that health, in the senses described, is a necessary part of all ten capabilities. Moreover, some of the capabilities on Nussbaum’s list, such as thinking and imagining, and practical reasoning, refer to health. Finally, it is shown that even though health is part of all capabilities, health cannot itself primarily be seen as a capability. An acceptable degree of health is required as a functioning for any theory of human flourishing to be reasonable.

## Introduction

Capabilities are conditions required for a person to do or be (or feel^1^) something (Sen 1979, 1995, 1999; Nussbaum 2000, 2011); they are his or her “substantial freedoms” (Sen in Nussbaum 2011, p. 20), or her or his *real (immediate, or practical*^2^*) possibilities*. A functioning is what a person (in fact) is or does (or feels; Ibid.; Sen 1995, 1999). The crucial distinction between a capability and a functioning is that in the first case the individual has a (real) possibility to do or be (or feel) something, and that in the other, she has realized the capability and is or does (or feels) this something. Some capabilities necessarily have to be turned into functionings, or the person cannot live a recognizably human life at all, and some functionings exclude others, that is, once a person has made a choice to actualize a functioning in a certain way, many others are thereby made impossible. For example, choosing to pursue one (full time) education (in general) excludes choosing to pursue another (at the same time); or if a person converts to Judaism, she cannot still be a Christian, or a Muslim.

Furthermore, capabilities are (in general) dimensions—first, in the sense that one can have many or few capabilities, and second (for most capabilities), in the sense that one can have more or less of each of them; one can, for example, have more or less of an ability or a skill, many or few educational options, or many or few possibilities to exercise one’s autonomous choices.^3^ In some environments people have vast amounts (as well as high degrees) of substantial freedoms, and in others less. In general, we might assume that many, or at least some, of the functionings that are actualized by people (at least in decent societies) are freely chosen, but only if the range of capabilities is rather large, since otherwise the person might have to realize functionings because there are no other options, for example, become a window cleaner, rather than a teacher, because of not being able to afford an education.

It is probable that in most cases the functionings that are “chosen” or actualized (given a range of options) are, at least instrumentally (Sen 1995, p. 41), important for the individual, and therefore contribute to the good life of the individual (Ibid, p. 39, ff.; Robeyns 2005, p. 95). The primary (political) point of the theory is that capabilities are of crucial importance for people (at least in liberal democracies) and that they (and their distribution) should be taken into account when assessing if life is good for people, or if the government of a state (or a municipality, or county council) is successful in creating the foundations for a good life for all (Sen 1995, 1999). According to Martha Nussbaum, “[c]apabilities, not functionings, are the appropriate political goals, because room is thereby left for the exercise of human freedom” (2011, pp. 25–26), and “freedom has intrinsic value” (Ibid. p. 25).

The factors that together constitute capabilities can be divided into those that are internal to the individual, and those that are external to her. One of the internal factors is, it seems, health. The value and importance of health is emphasized by Amartya Sen in many publications (for example, in 1979, 1995, 1999, and 2004). He does not, however, define what he means by health. Neither does Sen develop a more detailed list of capabilities, even if we can assume that he sees health as important for the capabilities. Nussbaum, on the other hand, is more specific about the capabilities and has suggested a list consisting of ten capabilities that she finds especially important, and perhaps exhaustive (2000, p. 70 ff., 2011, pp. 33–34).^4^ However, even if her list of capabilities includes health, she does not develop a theory of health either, nor does she provide a definition of the concept.^5^

The aim of this paper is to make sense of health in the light of Nussbaum’s version of the capability theory, that is, determine what we should mean by health, and what its relation is to the overall theory.

## What can be part of a capability

Before discussing health, and then health in relation to the capabilities, it might be a good starting point to present, on a fairly abstract level, what can be part of a capability, or rather, what Nussbaum calls *combined capability*.^6^ As we have already seen, a capability consists of all those internal and external factors that are necessary for a specific case of doing or being (or feeling) something. Lacking a capability is lacking a sufficient degree of at least one factor that is necessary for actualizing the functioning in question.

Here are some suggestions as to what a capability might include as constitutive parts.^7^ First, the internal factors:

- *Basic abilities*, that is, abilities that are developed without special education or training. These abilities are either “intentional”, such as the ability to stand up, lie down, walk, grab, reason, chew, and talk, or “innate” (or automatic), that is, not (in general) under the conscious control of the individual, such as (being able) to see, hear, smell, taste, and keep one’s balance.
- *Basic dispositional traits*, for example, resilience to stress, self-esteem and self-confidence, impulse control, and being able to experience emotions.
- *Competences* (that is, advanced abilities that usually require education or special training^8^), such as work-related ones, for example, being able to use advanced computer software, or master a bulldozer, and non-work-related ones, for example, being able to cook, or ride a bicycle. Competences can be divided into knowledge and skills (Ryle 2000; Nordenfelt 2008). Note that competences require (at least a minimal degree of) some of the more basic abilities and dispositions mentioned above.
- *General psychological motivation*,^9^ for example, wanting to get up in the morning and go about things. Motivation is a general prerequisite for being able to use one’s acquired abilities and competences.

Foundational for most of those internal conditions are:

- The physiological, anatomical, and (deep) psychological states and processes required for the abilities and competences mentioned, for example, that one’s joints work, one’s inner organs do what they are supposed to do, and one’s neural network is in order, that is, the brain’s functions are, for example, efficient, rapid, durable, and reliable (Pestana 1998).

There are, furthermore, internal factors that contribute to the realization of capabilities but that do not seem to be all that necessary for them.^10^ Some causally contributing factors might, for instance, be:

- Various kinds of subjective well-being (and freedom from suffering).
- Self-knowledge, for example, being aware of one’s strengths and shortcomings.
- Virtuous character traits, for example, being friendly, courageous, industrious, or honest.
- Morality (other than ability for it, which is part of the basic abilities), for example, following expected moral principles (that is, behaving in ethically appropriate ways).
- Personality and temperament.

Note that most of those abilities and competences, etc., come in degrees. In general, the more one has of them, the better (one’s capabilities). However, certain capabilities might only require a limited number of them, and a minimal degree of (some of) them. For example, the capability to “take a walk in the park” requires certain basic abilities (such as the abilities to walk and perceive), those abilities’ physiological, anatomical, and psychological foundations, some motivation, knowledge about where the park is located (and how to get there), and, of course, the (external) opportunity to take a walk in the park (for example, a safe and accessible park to walk in, and some free time).

As to the external aspects of a capability (that is, opportunity), I will be briefer, since they are not (in general) regarded as belonging to health. We have:

The physical environment:

- A supportive natural environment.
- Acceptable weather and climate conditions.^11^
- Infrastructure, energy, and technology.
- A home, domestic appliances, tools, and utensils.

The social environment:

- Negative and positive freedoms (which include some of the factors below).
- A tolerant (or liberal) political, economic, and legal framework for action or being.
- Norms supportive of the action (or non-action) or being (or feeling) in question.
- Personal security.
- Work opportunity, money, and other social assets.
- Psychological (and other kinds of) support from partner, family, friends, and colleagues.

Some of the factors listed (at least partly) cover Sen’s lists of five instrumental (social) freedoms: political freedoms, economic facilities, social opportunities, transparency guarantees, and protective security (1999, p. 10).

All those factors (internal or external) are, of course, not equally supportive of (or necessary for) all capabilities. An investigation into the person’s aspirations and situation will tell us more about what is, in the specific case, necessary for doing or becoming (or feeling) something.

## The boundaries of health

Where should we draw the boundaries of health? Within the individual, say some (Whitbeck 1981; Fulford 1989; Nordenfelt 1995, 2016; Brülde 2000a, b; Tengland 2007). So that it includes parts of the environment as well, say others (Pörn 1984; Seedhouse 2001; Venkatapuram 2011). The former idea seems more plausible. A major drawback of including the environment in the concept is that it becomes impossible to differentiate between health and its determinants. Let us say that drinking water, part of our environment, is also part of a person’s health. But drinking water is also a determinant of health (and survival). Drinking water is thus, on this view, both part of health and its determinant, which appears contradictory. We can conclude, then, that none of the factors listed above that are external to the individual should *be part* of an individual’s health.^12^

There is still the difficulty of deciding exactly what within the individual belongs to health, since everything internal is not part of an individual’s health either. Before trying to answer this question, we first have to ask what we need a theory (and a definition) of health for. This is mainly of practical importance (Brülde 2000a, b; Brülde and Tengland 2003). In society we need to establish professional boundaries and governmental responsibilities. Health is one responsibility, education is another, and creating work opportunities a third. Cutting the cake (conceptualization) can be done in various ways, but one way might still be superior to another (given our practical needs). So one important question is what view of health makes most sense given the division of labor that we have in our societies, where professional and institutional areas, such as medicine, health care, and public health, have certain responsibilities (for example, to treat, cure, and prevent ill health, and promote positive health).

One criterion for a successful theory (and definition) of health is, then, that it has practical relevance. To this we can add that the theory (and definition) produced should be in reasonable accordance with ordinary language use (descriptive adequacy), that is, how people speak about health and ill health. There should, of course, be a rather high agreement between this criterion and the professional division of labor, even if they might not match perfectly. Two prominent aspects found in common parlance regarding health (and ill health) are, suggests Lennart Nordenfelt (1995, 2016), ability and (subjective) well-being (and its opposites disability and suffering). This is in agreement with what central parts of medicine and health care deal with (for example, the restoration of ability and the alleviation of suffering) and health promotion tries to achieve. Not all kinds of ability and well-being are, however, health related, so some qualifications have to be made in order to make the definition more precise. There is, then, a stipulative element in choosing these as key components of health, but the stipulations can be, and have been, defended (see Nordenfelt 1995, 2016; Brülde and Tengland 2003; Tengland 2007).

Finally, it is of moral importance how we define politically significant concepts.^13^ It is thus an advantage if the suggested theory of health increases our ability to make ethical judgments, for example, in (a Western context) deciding whom to help and when: helping someone with a health need in general seems more morally pertinent than helping someone with financial problems (assuming that the financial problems are not the immediate cause of the ill health). In relation to this we might add that even if capabilities, and indirectly quality of life, might be the ultimate political value, and society therefore should see to it that the population acquires a high degree of them (and that they are fairly distributed), it seems that understanding what health is, is politically crucial. The reason is not necessarily that health has final value, which it only partly has, but rather that health, as I will argue, is such an important part of the capabilities.

Let us return to the list suggesting internal conditions for capabilities (on page xx). Based on the above reasoning (and similar reasoning elsewhere; see Tengland 2007), that is, combining our linguistic intuitions and the importance of drawing professional boundaries, with the ethical aspects, health should consist in (at least) an individual’s *basic (intentional* and *innate) abilities* and *dispositions*,^14^ a certain kind of *subjective well*-*being*,^15^ which will be referred to as *manifest* (or holistic) health, and those more *fundamental internal states and processes* that are required for upholding manifest health, which will be referred to as *fundamental* health (Tengland 2010, 2015; see also Brülde 2000a, b). Moreover, some kind of basic *motivation* seems to be part of health, since without it no action would take place at all. These are precisely the kinds of internal features of people that health promotion and public health want and ought to promote and protect, and the kinds of states and processes that medicine and health care treat and take care of when they have been reduced.

Those internal features of a person (listed above) that are not part of her health are, then, her knowledge (including her self-knowledge), skills and competences, her virtues, her morality, and her personality and temperament.^16^ Those aspects of a person can, of course, be reduced because of ill health, as when a person cannot ride a bike (a competence) because she cannot grip the handlebars (basic ability), due to a fractured wrist (an injury). More could be said here, but this is hopefully sufficient for the rest of the discussion.

## Nussbaum and capabilities

Let us now turn to Nussbaum’s list of capabilities (2000, 2011). It is well known, so I will be brief. The capabilities (here abbreviated) are: “[b]eing able to” (2011, pp. 33–34)

1. live a normal life span,
2. have good bodily health, including reproductive health,^17^
3. experience bodily integrity, freely move around, and make reproductive choices,
4. use one’s senses and imagination, and think,
5. experience emotions and have emotional attachments,
6. exercise one’s practical reasoning in order to form a conception of the good, and critically reflect about one’s life choices,
7. establish affiliations, that is, live with others and have a social basis for self-respect,
8. live with, and express concern for, other species,
9. play, laugh, and enjoy recreational activities, and
10. participate effectively in political choices, and control one's social and physical environment, including to hold property and seek employment.

However, Nussbaum also presents the idea of “internal capabilities” (Nussbaum 2011, p. 21). They consist of a variety of “fluid and dynamic” states, such as personality traits, skills, knowledge, capacities, and health (Ibid., p. 21).^18^ She goes on to add what she calls “basic capabilities” (Ibid., p. 23), those innate physiological, anatomical, and (deep) psychological factors that “make later training and development [of the internal capabilities] possible” (Ibid., p. 24). Whereas the internal capabilities have to be trained (Ibid., p. 21), as when a skill is exercised, the basic capabilities need to be “nurtured” (Ibid., p. 23), for example, through a regular intake of nutrition.

Note that Nussbaum’s mentioning of health, as part of a person’s internal dynamic states, fits well with this paper’s suggestion that (manifest) health consists in a person’s basic abilities (and dispositions) and subjective well-being (both clearly internal dynamic states). She is also careful to differentiate health from a person’s other internal states, such as skills and knowledge, as is also done in this paper. The difference, then, compared to the theory proposed here, is that Nussbaum does not specify which of an individual’s internal states belong to health and how they are differentiated from the other dynamic states.

## Nussbaum and health

Having decided on a theory of (manifest and fundamental) health, I will now examine what the relation is between the items on Nussbaum’s list and health.

Health is the second item on her list. In a footnote, Nussbaum (2000), when discussing reproductive health, seems to adopt the WHO idea that health is “complete physical, mental and social well-being” (p. 78, footnote 83). This idea has been widely criticized, however, both for being too broad, for example, by including states of happiness, or quality of life, in the definition of health, and for being vague regarding what “well-being” stands for (Callahan 1973; Brülde and Tengland 2003, pp. 237–238). It also excludes an important aspect of health, namely, ability (Nordenfelt 1995).^19^ So, the capabilities theory requires something better.^20^

Before we get to the discussion about some of the capabilities and their relation to health, a theoretical issue has to be dealt with. When Nussbaum presents her ten capabilities, she consistently writes “[b]eing able to…” (2011, pp. 33–34). Ability in this context, it should be clear, encompasses opportunity, since we are talking about “combined capabilities”, which consist of both internal and external conditions (Nussbaum 2011, pp. 20–21). Now, there are two ways of interpreting this way of formulating the capabilities, that is, as “being able to”. One is that people should be able (and have the opportunity) to *acquire* the capability in question. The other is that the person should be able (and have the opportunity) to *exercise* (or actualize) it (here and now). The difference is between, for example, being able to learn to read (here and now), and being able to read (here and now), or between being able to acquire an education (for a specific profession), and work in (exercise) the chosen profession. In Nordenfelt’s terms, the first would be a “second-order ability”, and the second one a “first-order ability” (1995, pp. 49–53).

Concerning health, the first interpretation (second-order) would be that the person should be able to acquire health (here and now), and the other (first-order) one that she should be able to “exercise” her already acquired health (or *sustain* it, as I will later suggest). The second-order view of ability seems less attractive, especially concerning health.^21^ It does not seem reasonable only to require that people should *be able to become* healthy (if they so wish). Rather, people *should have acquired* health (at least when they come of age).

Besides explicitly placing health on her list of capabilities, Nussbaum also includes several other “abilities” (*the internal parts of*) which, I would argue, belong to health, since they belong to the person’s basic abilities and dispositions that I have suggested are part of manifest health. *Sense*, *imagination*, and *thought*, the internal parts of capability four, are all basic mental abilities, the reduction of which should be the concern of psychiatry, medicine, and health care. For example, a reduced ability to think clearly, something that might be the effect of dementia, appears to be such a responsibility. Capability five also seems to include health-related aspects. *Being able to experience emotions*, and *being able to form emotional attachments*, are typical basic dispositions and abilities that people acquire in most societies, and the absence or reduction of either of them seems to be a sign of reduced mental health. The state of alexithymia (Taylor 2000), that is, not being able to describe, or identify, emotions, for example, is considered a case for psychiatry, or neurology. *Practical reason*, the sixth capability, (the internal part of) which, I take it, is (more or less) synonymous with the ability for autonomy, also, I would argue, belongs to the basic abilities. Having a reduced ability for self-determination, a possible effect of, for example, substance abuse, is having reduced mental health, and such a condition is arguably a responsibility for health care. It is probable that the capabilities mentioned in this section should also be interpreted in the first-order sense, that is, what matters is being able to “exercise”, not being able to acquire, them.

Finally, the first capability on Nussbaum’s list, that is, live a normal life span, is also clearly related to health. Living a normal life span presupposes that the individual is, at least, minimally healthy, but health is also instrumental in staying healthy over time (more on this later).

## The capabilities reconsidered

Both Sen and Nussbaum focus on the provision and distribution of capabilities. They should be “the currency of egalitarian [social] justice” (Cohen 2011, p. 3; Sen 1995, 2009). However, whether or not these capabilities are turned into functionings is a matter for each individual. The important thing, politically and morally, is that (at least a minimal degree of) any one of the ten capabilities can be actualized at any time (assuming that a functioning does not exclude others from being exercised at the same time). Nussbaum claims that “it is capabilities, not actual functionings, that should be in [*sic*] the legislator’s goal” (1990, p. 224). The relative lack of (theoretical) interest in what functionings people do in fact acquire, has, as we have already seen, to do with Nussbaum’s (and Sen’s) liberal political orientation. No one else should have the right to determine how people should live their lives, that is, what to be or do (or feel).

This focus on capabilities might sound more convincing (to a liberally oriented person) than it is. I want to argue that some functionings, not only capabilities, *should* also be regarded as “the legislator’s goal”, and that health is one such functioning. First, it seems clear that certain functionings, and especially health (in the above-mentioned senses), are presupposed by, and are, therefore, part of all capabilities.^22^ Some degree of health, especially mental health, is required for all functionings (and is, thus, part of all capabilities), such as to freely move around (which requires the abilities to stand, walk, and hold one’s balance), to play (which often requires the basic abilities to move and imagine), establish affiliations (which requires the disposition to experience emotions), make reproductive choices (which requires the ability to think and deliberate), live with other species (which requires basic perceptual abilities), control the environment (which requires critical reflexivity), seek employment (which requires basic psychological motivation), and make political choices (which requires basic reasoning capacity). Thus, (some degree of) health, *as an actuality* (functioning), is necessary for any kind of activity and, thus, for living a “normal” life.

Second, this also means that the list of capabilities needs some clarifications and amendments. Health, the second item on Nussbaum’s list, should not itself be seen as a capability (except, as was just claimed, as a constitutive part of all capabilities). It is not a second-order ability that can be acquired, if so chosen, nor an “ability” *waiting to be exercised*, as when a person who has learnt to read chooses to read, or as when a person has an education (and, thus, a competence waiting to be exercised) and then applies for a job, gets it, and starts working. It is not as if the (health-related) abilities to deliberate, move around, perceive, and think await a decision for them to be realized. The health-related abilities and dispositions *are* present and constantly realized, that is, they are functionings.

Thus, every individual, at least when she comes of age, has to *be* equipped with a decent degree of health, in order for us to be able to say that she is well prepared for (a good) life.^23^ Health, then, is a functioning that people should have acquired and “use” (when they come of age) in order for a society to be considered decent, and not a capability that can be actualized (or exercised), if the individual so chooses.^24^

Having secured this necessary functioning, we need to make sure that people have the capability to maintain it. This, it seems, is (at least for a liberal) where freedom, or choice, enters, that is, choosing to, or not to, *sustain* the functioning.^25^ Thus, including (general) health (as a specific item) on any list of capabilities should probably only mean that the (hopefully already healthy) individual has a real possibility to *remain healthy*.

Furthermore, some of the other capabilities (or parts of them) mentioned in the list, are, as we have seen, part of (general) health. As they are part of health, they, of course, also have to be functionings. In order to “exercise one’s practical reasoning” and to “critically reflect” (given the opportunity) the person should already have acquired a high degree of the ability for autonomy (as a functioning). The capability being able to “use one’s senses, imagination, and think” is, as we saw, also health related, since sensing, imagining, and thinking are all basic abilities. But they obviously also go beyond health, since these basic health-related abilities can be developed in various ways (that is, be developed into competences), which shows that health in this sense (basic cognitive abilities) is presupposed for the more advanced capability to think. The generic ability to imagine (health) can be used for writing a novel (a skill), and thinking (health) can be trained to encompass advanced logical reasoning (a skill). Thus, having the real possibility to think advanced thoughts, and to imagine creatively, already requires several basic (health-related) abilities and dispositions as functionings. My point is that these specific (health-related) examples from Nussbaum’s list should all be considered necessary functionings, and the related capabilities are (as for general health) about sustaining those health-related functionings. To be upheld or sustained, they also, among other things, require that the individual is (minimally) healthy, for example, is able to exercise, is able to understand health information, and is able to make informed and rational decisions concerning health. In brief, the capability (suggested in this paper) to stay healthy (sustain health) and Nussbaum’s first capability to live a normal life span require (some degree of) health.

What has been said about (manifest) health, namely, that (some degree of) it is required for all capabilities, also (and even more so) pertains to what Nussbaum has called “basic capabilities”, that is, those internal factors that make training and development possible and that, as I have suggested, constitute the person’s fundamental health (Tengland 2010, 2015). Basic capabilities (that is, fundamental health) should obviously not be called capabilities at all, but *basic functionings*, since they are necessary (to a high degree) for all of the health-related abilities and dispositions, for (health-related) subjective well-being, and for most competences, as well as for living a normal life span. Thus, if the internal states and processes do not function as they are supposed to do (at least to some minimal degree) we cannot act, and will not survive for long.

In contrast to manifest health and fundamental health, which should be seen as necessary functionings, there is nothing necessary about capabilities such as being able to express concern for other species, enjoy recreational activities, or participate effectively in political choices. They can be actualized (exercised), or not, and nobody would be (really) alarmed if they were not (even though we might think that the individual would be better off acting on them). Furthermore, they are not part of, or required for, actualizing (exercising) the other capabilities. A person could go through life indifferent to politics (at least if she lives in a decent society, where others are politically active) or to other species, and still actualize (exercise) most other capabilities, that is, turn them into functionings.

## Conclusion

This close look at the role of health in the capabilities shows why Sen and others are correct in emphasizing the value and importance of health for living a flourishing life. In the paper, I have argued that (some degree of) manifest health is a constitutive part of all the capabilities, that is, that some basic abilities and dispositions, etc., are necessary for any capability to be minimally present.^26^ Moreover, there are internal requirements for manifest health to be possible, namely, that a person’s anatomy, physiology, and “deep” psychology support it. This level, that Nussbaum calls “basic capabilities”, is, I have suggested, an individual’s fundamental health, that is, a functioning, and not a capability at all (since its realization is necessary in order to live and function holistically). I have, furthermore, argued that many of Nussbaum’s capabilities already “illustrate” (or encompass) various aspects of health, such as the ability for autonomy, sensing, imagining, and thinking, and being able to experience emotions.

Moreover, providing a list of capabilities is not sufficient, theoretically or politically, since there are capabilities that are so fundamental that they *have to* be actualized, that is, be functionings, for us to be able to say that a life goes well. A list of functionings thus has to be added as a complement to the list of capabilities, which in turn should be revised in order to make better sense of this addition. Health (in its various aspects) would be on this list of functionings. Any lack of this functioning (at least when coming of age) implicates that society has failed.^27^ However, the real possibility to *sustain* health over a lifetime (which also covers the first capability, “to live a normal life span”) should still belong to the list of capabilities.

## Footnotes

1 This addition was suggested to me by Bengt Brülde. Personal communication 2017.

2 The term “practical possibilities” is used by Lennart Nordenfelt (2000, p. 65).

3 Note, however, that some functionings are binary, e.g., being married or not, or require mutually exclusive choices, as exemplified above.

4 Other capabilities have been suggested. See, for example, Wolff and De-Shalit 2013.

5 Neither does Jennifer Prah Ruger, who has written extensively about health and social justice (in a capabilities context), provide a definition, other than making vague references to health functioning (2010a), which is neither “well-being” nor “quality of life” (2010b, p. 42). Iain Law and Heather Widdows (2008) have also written about health and capabilities, but they have not yet provided (as far as I can see) their promised alternative theory concerning the relation between capabilities and health (p. 311, footnote 3). Sridhar Venkatapuram has, however, developed a theory of health, in relation to the capabilities theory. For a discussion of his theory, see Tengland 2016, where I initially introduced some of the ideas developed here.

6 I will, however, stick to writing “capability”, since all capabilities have to be combined ones, that is, every being and doing (and feeling) requires both (internal) ability and (external) opportunity (see Nussbaum 2011, p. 20).

7 Meaning that a fair (sometimes a considerable) degree of some of these factors is required for the capability to exist.

8 As one reviewer noticed, it is not always possible to make a clear distinction between basic abilities (health) and competences, since the former can become quite advanced over time (without needing “education or special training”).

9 Motivation, here, is about being able to form intentions and (want to) act on them (Nordenfelt 2006, p. 1463). Some might prefer to call this energy, drive, or will.

10 In reality, the total absence of some of these internal features will compromise a capability, for example, when severe pain (suffering) makes any kind of action impossible, or when lack of self-knowledge makes a person choose poorly.

11 “Unacceptable” weather and climate conditions can usually be compensated for in affluent societies.

12 Note, however, that the environment is important for a theory of health in another way. The environment makes actions possible (and sometimes impossible), and is a “platform” for action. Thus, to be healthy is to be able to act (or pursue one’s goals) given that *the environment is acceptable*, which means that in it people are expected to be able to act (or pursue their goals). See Nordenfelt 1995 (pp. 47–49), for an explication of this idea. For a detailed conceptual discussion of the boundary between what is external (environment) and internal (in relation to health), see Tengland 2015.

13 For a thorough analysis of the concept, we need more criteria, but these, I believe, will suffice for the present purposes.

14 Note that the individual should not only have acquired the abilities (etc.) in question, but also be able to use them here and now (given that the environment is acceptable), in order to be considered healthy.

15 Briefly, health-related, subjective well-being (and suffering) is those kinds of sensations and moods that have their immediate cause within the person (Tengland 2007, 2016), for example, feeling fit, or experiencing pain. Much more will not be said about subjective well-being, since this aspect of health is less important for the capabilities than abilities.

16 This claim might have to be qualified. See Tengland 2012, for a discussion of morality and health.

17 It is unclear why mental health is not included here. My use of the concept of “health” in discussing this capability, later in the text, will cover both physical and mental aspects. However, some of Nussbaum’s other capabilities include aspects of mental health, as I will argue.

18 Thus, Nussbaum uses the term health in two different ways in her theory, as a capability and as part of an internal capability, and it is unclear how they are related.

19 Note that in the Ottawa Charter for Health Promotion (WHO 1986), and later WHO charters, health is seen as a “resource for everyday life” (p. 1). This resource is “personal and social”, and includes “physical capacities” (p. 1). This is something quite different from (total) well-being. This idea is, however, still partly unclear, but one reasonable interpretation of it is that what the WHO calls resources are what some theoreticians call abilities (or capacities).

20 Nussbaum’s second capability, health, could also be interpreted to mean being able to stay free from disease, as Venkatapuram at one point suggests (2011). As such it is an obvious capability. However, we can still require (morally and politically) that the person who has this capability also *be* free from disease (a functioning), insofar as it is possible.

21 And it is probably not the view intended by Nussbaum, even though my discussion of it (Tengland 2016) in relation to Sridhar Venkatapuram’s theory (2011) made it appear a second-order one. Note, however, that for some kinds of capabilities the second-order ability might suffice. A person might, for example, want to work as a teacher, but not be able to because she has not got the appropriate education (and, thus, no first-order ability to do so, even if all other conditions needed are present). Nevertheless, the person can still have the second-order ability to acquire the education that permits her to work as a teacher. In such a case the second-order ability to work seems to suffice for us to be able to say that the capability (to work as a teacher) is present (assuming that all other conditions for it are satisfied).

22 Ruger makes a similar claim when she notes (in passing) that “certain aspects of health are prerequisites for other types of functioning” (2010a, p. 3). She does not, however, specify in what respect. Sen made the same observation in 2004 (p. 23).

23 This, as Nussbaum recognizes (in relation to children; 2011, p. 26), also goes for other skills and competences, including those learnt in primary and secondary school, for example, (generic skills such as) to write, read, count, cooperate, plan ahead, solve problems, and communicate.

24 This assumes that the individual has an internal potential to be fully healthy, and is not, for example, born with a dysfunction of some kind.

25 This means that, if people live (“freely chosen”) lives that reduce their health, many of their capabilities are likely to be reduced over time as well. Therefore, no (liberal) government can guarantee a minimal degree of capabilities for all (adults), over time.

26 And a certain degree of (health-related) subjective well-being (or at least absence of suffering) will contribute to the capabilities, that is, subjective well-being makes actions and goal attainment easier, whereas suffering makes things more difficult.

27 Assuming, once again, that the individual has the required inborn potential to develop these abilities and dispositions, and experience (health-related) subjective well-being.
